# Transcriptomics of liver and muscle in Holstein cows genetically divergent for fertility highlight differences in nutrient partitioning and inflammation processes

**DOI:** 10.1186/s12864-016-2938-1

**Published:** 2016-08-11

**Authors:** Bruce Moran, Sean B. Cummins, Christopher J. Creevey, Stephen T. Butler

**Affiliations:** 1 Teagasc, Animal & Grassland Research and Innovation Centre, Grange, Dunsany, Co. Meath, Ireland; 2 UCD Conway Institute of Biomolecular and Biomedical Research, University College Dublin, Belfield, Dublin 4, Ireland; 3 Teagasc, Animal & Grassland Research and Innovation Centre, Moorepark, Fermoy, Co. Cork Ireland; 4 Institute of Biological, Environmental and Rural Sciences, Aberystwyth University, Aberystwyth, SY23 3FG UK

**Keywords:** Gene expression, Dairy cow fertility, Liver, muscle, Lactation

## Abstract

**Background:**

The transition between pregnancy and lactation is a major physiological change for dairy cows. Complex systemic and local processes involving regulation of energy balance, galactopoiesis, utilisation of body reserves, insulin resistance, resumption of oestrous cyclicity and involution of the uterus can affect animal productivity and hence farm profitability. Here we used an established Holstein dairy cow model of fertility that displayed genetic and phenotypic divergence in calving interval. Cows had similar genetic merit for milk production traits, but either very good genetic merit for fertility traits (‘Fert+’; *n* = 8) or very poor genetic merit for fertility traits (‘Fert-’; *n* = 8). We used RNA sequencing to investigate gene expression profiles in both liver and muscle tissue biopsies at three distinct time-points: late pregnancy, early lactation and mid lactation (-18, 1 and 147 days relative to parturition, respectively).

**Results:**

We found 807 and 815 unique genes to be differentially expressed in at least one time-point in liver and muscle respectively, of which 79 % and 83 % were only found in a single time-point; 40 and 41 genes were found differentially expressed at every time-point indicating possible systemic or chronic dysregulation. Functional annotation of all differentially expressed genes highlighted two physiological processes that were impacted at every time-point in the study, These were immune and inflammation, and metabolic, lipid and carbohydrate-binding.

**Conclusion:**

These pathways have previously been identified by other researchers. We show that several specific genes which are differentially regulated, including *IGF-1*, might impact dairy fertility. We postulate that an increased burden of reactive oxidation species, coupled with a chronic inflammatory state, might reduce dairy cow fertility in our model.

**Electronic supplementary material:**

The online version of this article (doi:10.1186/s12864-016-2938-1) contains supplementary material, which is available to authorized users.

## Background

The transition from late pregnancy to early lactation in dairy cattle has important implications for animal health, milk production and reproductive performance, and consequently the profitability of dairy farms [1, 2]. The physiology of the transition dairy cow has been well studied; a variety of biological themes such as energy balance, body reserve mobilisation, metabolic diseases, insulin resistance and immune function have all been found to be of importance [3–8]. Transcriptomics has been employed to aid identification of genes and networks that impact these various systems [9–11].

Hepatic gluconeogenesis, which supplies the vast majority of glucose utilized by ruminants, is of central importance in dairy cattle. The liver uses propionate (60–74 %), L-lactate (16–26 %) and other minority precursors for glucose synthesis [5, 12, 13]. During late pregnancy, the glucose demands of the fetus account for up to 50 % of total maternal glucose production [12]. During early lactation, high-producing dairy cows have a greater demand for glucose than can be met by gluconeogenesis from dietary sources alone. For example, dry matter intake (DMI) increases during the transition from late pregnancy to early lactation by 30 to 50 %, whereas mammary glucose requirements during early lactation are three times that of the uterus during late pregnancy [14]. Consequently, dairy cows enter negative energy balance (NEB), resulting in body reserve mobilisation. Insulin-sensitive tissues such as adipose and muscle become insulin resistant coincident with reduced circulating insulin concentrations. The combined effect of these changes is reduced peripheral tissue uptake of glucose, and hence greater glucose availability for the mammary gland. Local energy requirements in some tissues, for example muscle, can be met by the use of non-esterified fatty acids (NEFA) from adipose tissue lipolysis; the associated release of glycerol can be used by liver for gluconeogenesis [15].

Homeorhesis requires orchestrated changes across multiple tissues to support prioritization of nutrients for lactation [12, 16]. The task of elucidating the physiological mechanisms underlying transition cow homeorhetic adaptations is ideally suited to transcriptomic analysis. Multiple tissues can be biopsied at multiple time-points to allow investigation of the genes involved in functional pathways and inter- and intra-cellular signalling. A lactating dairy cow genetic model of fertility has been developed and validated [17–19]. Cows have similar genetic merit for milk production traits, but either good (Fert+) or poor (Fert-) genetic merit for fertility traits. The physiological differences between the cows in this animal model have been extensively characterized [17–22]. We have taken the approach of sequencing the transcriptome of liver and muscle biopsies collected from Fert + and Fert- cows at three time-points corresponding to late pregnancy, early lactation and mid-lactation. Using bioinformatic methods to interrogate these data, we test the hypothesis that gene expression differences exist between cows with good or poor genetic merit for fertility traits during late pregnancy, early lactation, and mid-lactation in these tissues. In particular, the latter time-point coincides with the stage of lactation when pregnancy should be re-established and so is of economic importance. Although liver and muscle are not reproductive tissues, they are both important tissues for determining bioenergetic status, glucose production and utilization, and body reserve mobilization and accretion. Collectively, these physiological processes have implications for phenotypic fertility performance. Hence, transcriptomic profiling of liver and muscle tissue in cows genetically divergent for fertility traits could aid identification of physiological processes that underpin differences in phenotypic fertility performance.

## Methods

### Lactating dairy cow model of fertility

The cow model used in this study has been described previously [17]. Briefly, the national dairy cattle database of Ireland was screened for heifers due to calve for the first time in spring 2008. Restrictions were placed on the estimated breeding value (EBV) for milk production (between +200 and +900 kg) and proportion of Holstein genetics (>75 %). Within this population, heifers with extreme positive (i.e., poor fertility) and negative (i.e., good fertility) EBV for calving interval were identified. The Fert + animals represented the top 20 % of the national herd in genetic merit for calving interval. Conversely, the Fert- animals represented the bottom 5 % of the national herd in genetic merit for calving interval. In 2009, 16 animals (*n* = 8 Fert + and *n* = 8 Fert-) were enrolled in the current study. Animals were selected to maximize genetic diversity within both strains (i.e., different sires and maternal grand-sires) and to maximize differences between strains in the EBV for calving interval. In both Fert + and Fert- groups, the cows were a mixture of first (*n* = 2) and second (*n* =6) parity Holstein animals (mean proportion of Holstein genetics (± SD) = 0.93 (±0.05)), and were managed as a single herd.

### Animal characteristics

The experimental procedures involving animals on this study were approved by the Teagasc Animal Ethics Committee and licensed by the Department of Health, Ireland, in accordance with the Cruelty to Animals Act (Ireland 1876) and the European Community Directive 86/609/EEC. The animals were owned by Teagasc Moorepark, and all animals in the herd are routinely used for research purposes. Milk production was recorded daily, body weight was recorded weekly, body condition score was recorded every two weeks and blood samples were collected periodically during late pregnancy and throughout lactation for analysis of plasma insulin, insulin-like growth factor-1 and non-esterified fatty acid concentrations as previously described [17]. The data for these variables from the specific animals used in the current study are reported to aid interpretation of the animal performance and the transcriptomic results. The data were analysed using SAS version 9.3 (SAS Institute, Cary, NC). All data were tested for normality and log-transformed if necessary. Milk yield, bodyweight and plasma concentrations of insulin, IGF-1 and NEFA were analysed using mixed models procedures with repeated measures. A first-order autoregressive covariance structure was applied, and cow nested within genotype was included as a random effect. Genotype, week, and their interaction were included as fixed effects. The Tukey adjustment was included to correct for multiple comparison tests. The BCS data was analyzed using generalized mixed model procedures using a similar model, but because BCS data is ordinal, a multinomial distribution and a cumulative logit link function were specified. None of the animals were bred during the lactation period in which the samples we collected. Hence, there is no confounding effect of pregnancy status on any of the observed phenotypes.

### Tissue sampling and RNA extraction

Tissue biopsies were collected at three time-points relative to parturition (day 0): late pregnancy (LP), day -18 (sd = 7); early lactation (EL), day 1 (sd = 1; EL); and mid-lactation (ML), day 147 (sd = 13). Liver tissue was collected by puncture biopsy as previously described [18]. To collect muscle tissue, a biopsy site on the semitendinosus muscle was shaved and sanitized with 7.5 % iodinated povidone and methylated spirits. A subcutaneous injection of lidocaine hydrochloride (2 %) was used to anesthetize the area. An incision was made through the skin, and the biopsy instrument (Biopsy Punch 33–37, Miltex GmbH, Riethein-Weilheim, Germany) was used to remove a core of muscle tissue. The incision site was sutured and treated topically with Duphacycline aerosol (3.6 % oxytetracycline hydrochloride: Norbrook Laboratories Ltd., Newry, Northern Ireland). Both liver and muscle tissue biopsies were immediately rinsed in saline, blotted dry, snap frozen in liquid nitrogen and stored at -80 °C until RNA extraction.

Total RNA was extracted using a standard Trizol-based method. The tissue sample was weighed and 100 mg cut and homogenized in 3 ml TRI Reagent (Sigma-Aldrich, Dublin) until fully homogenized using a hand-held device. The homogenate was removed to sterile Eppendorf tubes (Eppendorf, UK) and incubated at room temperature (RT) for 5 min; samples were centrifuged at 12,000 × g for 10 min at 4 °C, and the supernatant was removed to new sterile tubes. Chloroform was added at 0.2x the volume of homogenate and incubated at RT for 3 min and samples were centrifuged at 12,000 × g for 10 min at 4 °C. Isopropanol was added at 0.6x the volume of supernatant, vortexed and centrifuged at 12,000 × g for 10 min at 4 °C to pellet the RNA. The supernatant was discarded, the pellet was washed twice in 99 % ethanol (Sigma-Aldrich, Dublin), and centrifuged at 7,500 × g for 5 min at 4 °C. The RNA was re-suspended in 50 μl nuclease-free water (Sigma-Aldrich, Dublin). A kit based protocol (RNeasy Plus; Qiagen, UK) was used to clean the total RNA, removing the fraction below 200 bp and any genomic DNA. RNA quality was assessed using the Bioanalyser 2100 (Agilent Technologies, UK) with the RNA Nano chip.

### Illumina library preparation, sequencing and alignment

Library preparation was conducted using the Truseq v2 kit (Illumina, UK) following the supplied protocol. The Bioanalyser 2100 (Agilent Technologies, UK) was used to visually determine quality of the libraries with the DNA Nano chip. Concentration was determined using the KAPA Library Quantification (KAPA Biosystems, USA) qPCR method to allow equimolar pooling of each library. Four pools of 24 libraries were made, the maximum possible based on the number of barcoded adapters available. Each pool was sequenced for 100 bp using a paired-end strategy across 3 separate flowcell lanes on an Illumina HiSeq 2000 (Illumina, USA) to minimize technical variation, resulting in 12 lanes of HiSeq data.

The quality of the sequence data was assessed using the FastQC package [23]. Before alignment, sequence ‘reads’ were trimmed using Trimmomatic with ‘trailing’ bases (i.e. those at 3′ end) under a Phred score of 30 being removed [24]. Alignment of reads to the *Bos taurus* Ensembl70 UMD_3.1 genome [25] was conducted using STAR aligner under default settings with the exception that ‘--outFilterMismatchNoverLmax’ was set to 0.02. This was because reads were trimmed dynamically to remove by Phred scores, reads were of different lengths and using the ratio of 0.02 mismatches per *x* bp meant reads were treated equally (version 2.2.0; [26]). Samtools ‘view’ method was used with the –f2 flag to return only primary alignments in proper pairs i.e. where the SAM flag was set to 83 and 163, or 99 and 147 for paired reads (version 0.1.18; [27]) and with the –b flag to convert from SAM to BAM files, required to construct count data. Before and after our primary alignment filtering, Samtools’flagstat’ was used to determine the numbers of reads aligning to the reference. Counts were determined using the featureCounts utility of the Subread aligner specifying that only reads with pairs on the same chromosome (flags -p, -P, -C), and with a maximum insert size of 590 kb (flag –D) could be used [28]. Counts were defined as the number of times a read-pair mapped to a single annotated feature (gene) of the genome.

### Pre-processing of count data

Count data from featureCounts were filtered before analysis to remove lowly expressed genes using an in-house method [29]. Firstly, all genes with no counts across all samples were removed. Samples were then split by condition and time-point (i.e. 8 samples per group) and calculations of fragments per kilobase per million reads (FPKM; [30]) were used to determine the level of gene expression. FPKM were calculated in R 3.0.1 statistical software [31] using the formula:

$$ \left({10}^9\times C\right)\hbox{---} \hbox{---} \hbox{---} \hbox{---} \hbox{---} \left(N\times L\right) $$

where *C* = normalized counts per gene, *N* = total counts per sample, *L* = gene length. The distribution of the mean of the logs (base = exp(1)) of the FPKM values (mean log(FPKM)) per gene in each condition was determined. Any genes found in the lowest 20 % in both conditions were removed from the dataset.

### Differentially expressed genes

#### Differential expression analysis

Filtered counts were used to determine differential expression (DE) of genes using EdgeR Bioconductor package [32, 33] under the common dispersion method. The count data was normalized using the trimmed mean of M (TMM) method in the EdgeR [34]. Gene expression differences between the Fert + and Fert- cows were determined at each time-point sampled (LP, EL and ML) resulting in 3 DE gene sets; these are called ‘contrasts’ in the EdgeR documentation [35]. A false discovery rate (FDR; [36]) of *P* < 0.05 was used to correct for multiple testing in all contrasts. Those genes found significantly DE were retained for further investigation.

#### Temporal differential expression profiling

To determine if genes found DE in one or more time-points had similar expression patterns across time-points (temporally), the logFC between genotypes at each time-point was used. If a gene was found DE at any time-point, logFC was tabulated to give an expression profile for the gene across LP, EL and ML. We indicated the temporal expression profile at each of the three time-points combined using 0 for not DE, and 1 or -1 for DE and up- or down-regulated in Fert + cows respectively. For example, a profile of 0,0,1 indicates not DE in LP, not DE in EL and DE up-regulated in Fert + in ML. A matrix was then constructed showing total genes per profile. From this we used a table to visualize numbers of genes with particular profiles. We refer to these as DE profiles in the following sections.

### Functional annotation

Genes found DE were used as input for the Database for Annotation, Visualization and Integrated Discovery (DAVID) [37, 38], which is a gene set enrichment method using a modified Fisher Exact test. For the ‘background’ gene set, we used the full set of genes for each contrast following the FPKM filtering step. ‘Functional Annotation Clustering’ analysis was conducted with default settings including the default *p*-value. The resulting table was downloaded. Each ‘Functional Annotation Cluster’ contains sets of genes with multiple, mostly similar, annotations (e.g., ‘cytoskeleton’ or ‘microtubule complex’) from databases such as the Gene Ontology [39], Interpro [40] and KEGG [41].

## Results and Discussion

### Characterization of the animals

**Fig. 1:**
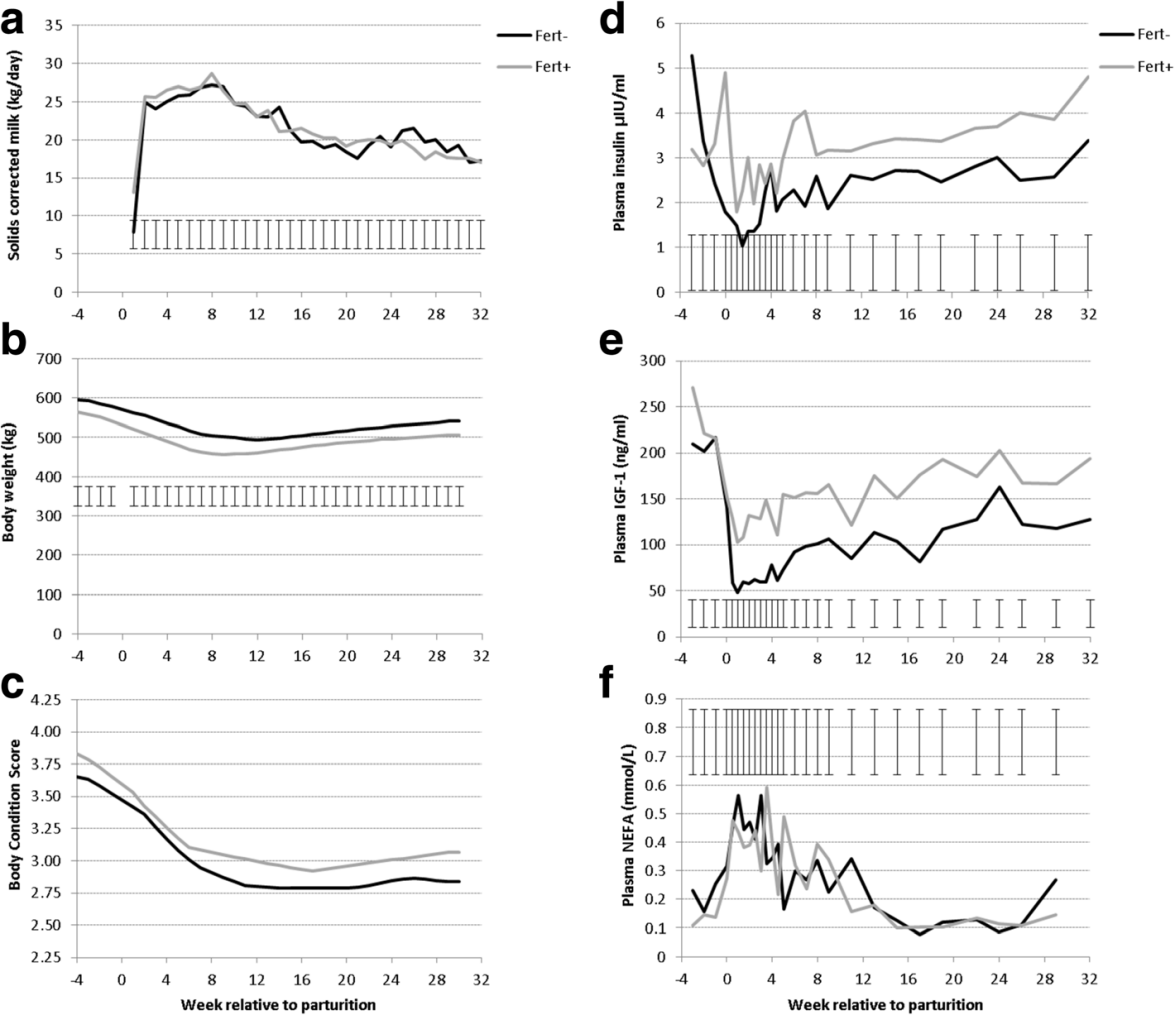
Phenotypic characterization of the Fert + and Fert- cows. Solids corrected milk yield (**a**) and bodyweight (**b**) were similar in both genotypes (P > 0.9 and P > 0.3, respectively), but BCS (**c**) was greater (P < 0.001) in Fert + cows compared with Fert- cows. Plasma insulin (**d**) and IGF1 (**e**) were greater in Fert + cows compared with Fert- cows (*P* = 0.003 and *P* < 0.001, respectively), but plasma NEFA concentrations (**f**) were not different (*P* = 0.9)

Milk production, bodyweight, BCS and plasma concentrations of insulin, IGF1 and NEFA are illustrated in Fig. 1. Milk energy output and bodyweight were similar, but Fert + cows maintained greater BCS throughout lactation. There was an overall effect of genotype on circulating insulin and IGF1 concentrations, but circulating NEFA concentrations did not differ. These phenotypes for the animals that had biopsies collected for the current study are similar to our previous reports with greater numbers of animals [17–19].

### Overview of differential expression

We found 402, 338 and 282 genes DE in liver and 262, 527 and 212 genes DE in muscle at LP, EL and ML respectively (Additional files 1 and 2). Functional annotation clustering analysis using DAVID identified annotation terms for 26 %, 25 % and 30 % of the total DE genes in liver and 39 %, 43 % and 44 % of the DE genes in muscle from LP, EL and ML, respectively (Additional files 1 and 2). Sequence data metrics are available in Additional file 3: Table S3.

### Temporal differential expression profiling

From the totals of 807 and 815 unique genes found to be DE in at least one time-point, 79 % and 83 % in liver and muscle, respectively, were up- or down-regulated at one time-point only (see Tables 1 and 2). In liver the top five DE profiles represented only a single time-point, and contained 12–18 % of all DE genes each. In muscle the two profiles with the most DE genes represented 25 % of all DE genes each, and were up- or down-regulated only at EL (DE profile 0,1,0 or 0,-1,0). This observation of time-point specific DE genes indicates that the time-points are relatively independent from each other in terms of DE gene profiles. Those DE genes found only at a single time-point are likely involved in distinct physiological processes specifically related to that time-point. This is not too surprising given that the time-points selected represent distinct phases in the cow’s gestation-lactation cycle.

**Table 1:** Liver temporal expression profile

			EL			
		0	1	-1		
		-	**144 (0.18)**	**94 (0.12)**	0	
	0	**41 (0.05)**	3 (0.004)	0	1	
		**106 (0.13)**	2 (0.002)	15 (0.02)	-1	
		**122 (0.15)**	15 (0.02)	2 (0.002)	0	
LP	1	41 (0.05)	23 (0.03)	0	1	ML
		11 (0.01)	1 (0.002)	2 (0.004)	-1	
		**129 (0.16)**	3 (0.004)	16 (0.02)	0	
	-1	0	0	0	1	
		19 (0.02)	1 (0.002)	17 (0.02)	-1	

All possible profiles across the three time-points and numbers of genes found with those profiles with proportion of total in parentheses; bold indicate profiles where only one time-point contained a DE gene

**Table 2:** Muscle temporal expression profile

			EL			
		0	1	-1		
		-	**202 (0.25)**	**207 (0.25)**	0	
	0	**30 (0.04)**	12 (0.01)	1 (0.001)	1	
		**80 (0.1)**	5 (0.01)	16 (0.02)	-1	
		**99 (0.12)**	23 (0.03)	3 (0.004)	0	
LP	1	11 (0.01)	17 (0.02)	0	1	ML
		2 (0.002)	1 (0.001)	0	-1	
		**53 (0.07)**	3 (0.004)	13 (0.02)	0	
	-1	2 (0.002)	0	0	1	
		11 (0.01)	0	24 (0.03)	-1	

All possible profiles across the three time-points and numbers of genes found with those profiles with proportion of total in parentheses; bold indicate profiles where only one time-point contained a DE gene

The remaining 21 % and 17 % of the DE genes in liver and muscle, respectively, were therefore found at more than one time-point. This may indicate processes that are systemic or chronic. Interestingly, 23 genes in liver and 17 genes in muscle were found to be up-regulated in Fert + cows at every time-point, and 17 genes in liver and 24 genes in muscle were found to be down-regulated in the Fert + animals at every time-point; these may represent dysregulated gene sets in Fert- animals.

Correlation plots of gene expression values in FPKM for each time-point versus the others to indicate concordance are reported in Additional file 4: Figures S1(a-f). Pearson correlation (R) value is included in each plot.

### Functional annotation

Two biological themes were apparent from the functional annotation of DE gene sets. The first theme, ‘*immune and inflammatory*’ processes, was identified from our annotation terms in both liver and muscle. This included time-point specific annotation terms ‘*chemokine*’ and ‘*MHC complex*’ in LP, ‘*defense response*’ and ‘*immunoglobuiln*’ in EL, and ‘*acute phase response*’ in ML. Of particular note, in this annotation multiple DE genes were found globally (i.e., across all time-points) and always in the same direction of expression (i.e., upregulated or downregulated in Fert + cows). This indicates that the identified genes (and the resulting annotation) are unlikely to be false-positives.

The second theme was ‘*metabolism, lipid and carbohydrate*’ related functions, including ‘*gluconeogenesis*’ and ‘*extra-cellular growth factor*’ in LP, ‘*biosynthetic process*’, ‘*lipid lipoprotein*’ and ‘*metabolic process*’ in EL, and ‘*lipid*’ and ‘*lipoprotein particle*’ at ML. There were also several interesting time-point specific functional annotation groups: ‘c*ell cycle/mitosis*’ in LP, ‘*tubulin*’ and ‘*tRNA*’ in EL in liver, and *‘actin/myosin*’ and ‘*tubulin*’ in EL in muscle.

### Global immune and inflammatory related gene annotations

Global differential expression of genes related to immune and inflammatory processes between Fert + and Fert- cows is a striking result in terms of reproductive efficiency. Genes down-regulated in Fert + cows included bovine leukocyte antigens *BOLA-DQA2* and *BOLA-DQA5* in both tissues as well as *BOLA* and *HLA-DQB2* in muscle. *BOLA-DQA1* and MHC class-1 *JSP.1* were up-regulated in muscle at every time-point in Fert + cows, and in liver both genes were up-regulated at both LP and ML. The ‘DQ’ genes are involved in pathogen-presentation to CD4(+) T-lymphocytes [42] and polymorphisms in these genes are involved in disease resistance in cattle [43]. While it was not possible to determine the cause of the immune responses observed, the allocation of resources towards these processes could have a systemic effect on the cows.

The solute carrier of carnitine, *SLC22A16*, was DE and down-regulated at all time-points in liver. Carnitine has recently been described as a general inflammatory response marker in dairy cows [44] and has been investigated in diet-induced inflammation. It may also be a marker for mitochondrial disease caused by increased circulating fatty acids in obesity [45]. Other mitochondrial-related genes were found globally down-regulated in liver, including cytochromes 7C (*COX7C*), *CYB5R2* and interferon-stimulated gene *ISG12(B)*, which may reside in the mitochondrial membrane and has been shown to mediate caspase-dependant apoptosis in humans [46]. One member of the caspase family, *CASP16* (a paralog of *CASP6*), was also found down-regulated. In muscle, three glutathione S-transferases, *GSTM2*, *GSTM3* and *GSTT1*, were globally down-regulated. These genes are activated by reactive oxygen species (ROS), and are necessary for clearing toxic oxidized glutathione from the cell [47]. This may be relevant to inflammatory processes, and therefore immune regulators are likely to respond.

### Time-point based assessment of annotations

#### Late pregnancy

##### ‘Immune and inflammatory’ related

An interesting group of genes found up-regulated at LP in liver included an ortholog of the complement factor H (ENSBTAG00000024647 found DE at approx. one fifth the expression level of *CFH*, ENSBTAG00000039995), CFH receptor 4 (*CFHR4*), which was the most abundant DE gene, and complement factor D (*CFD*). These genes represent an immunological response to disease, and also to the products of apoptosis and other cellular debris [48]. Differences in expression may indicate that Fert + cows have greater ability to regulate inflammation compared with Fert- cows at this timepoint.

##### ‘Metabolism, lipid and carbohydrate’ related

Functional annotation groups of the genes DE in liver during LP revealed the annotation term ‘*gluconeogenesis*’. This group included pyruvate carboxylase (*PC*), serine dehydratase (*SDS*), fructose-1,6-biphosphatase 2 (*FBP2*) and histidine decarboxylase (*HDC*), which were all down-regulated in Fert + animals (i.e., more highly expressed in Fert- animals). The role of *PC* is to convert pyruvate to oxaloacetate using lactate or amino acids (especially alanine, glycine, serine, cysteine and tryptophan) as a precursor, eventually resulting in glucose production. During periods of negative energy balance, the ‘labile protein reserve’ of skeletal muscle is a significant source of amino acids for hepatic gluconeogenesis [14]. This suggests that Fert- cows may have begun using body reserves for gluconeogenesis earlier than Fert + cows [5]. Further evidence for this comes from both the liver and muscle at LP.

The enzyme pyruvate dehydrogenase kinase 4 (*PDK4)* phosphorylates pyruvate dehydrogenase, thereby inhibiting conversion of pyruvate to acetyl coA. Greater tissue expression of *PDK4* has been implicated in switching to use of NEFAs for cellular energy requirements rather than use of pyruvate [49]. In our study *PDK4* was down-regulated in both liver and muscle (and was expressed at very high levels in the latter in Fert- animals, see Additional file 1: Table S1). It has been hypothesised that *PDK4* might be a ‘lipid-status’ responsive gene that facilitates pyruvate conservation. Increased *PDK4* expression mirrors lipid mobilization from adipose under starvation conditions [50]. This may indicate greater reliance on mobilised NEFA for cellular energy requirements in Fert- cows. In further support of this, fatty acid binding protein *FABP4*, which was up-regulated in Fert- cows in muscle, has been shown to have lipolytic activity in skeletal muscle [51]. Thus the increased *PC* and *PDK4* expression in Fert- animals may be the signature of muscle breakdown to provide amino acids for oxaloacetate production, as well as use of adipose and intramuscular fat for energy requirements. As this time-point is 3 weeks prepartum, however, it may be mistimed. The expression pattern observed indicates that Fert- cows might have initiated body reserve usage before Fert + cows, which may have a long term impact on their BCS further along in lactation.

At LP in muscle, we found the transforming growth factor-beta-inducible early growth response protein *TIEG1* up-regulated. This protein has been implicated in two key cellular processes: (i) apoptosis of cells damaged as a result of mitochondrial beta-oxidation of fatty acids during NEB; and (ii) inhibiting cell proliferation [49]. Other up-regulated genes with a functional role in this area include enoyl-coA hydratase *ECHDC2*, which is involved in the second step of beta-oxidation producing NADPH and acetyl CoA, and the fatty acid desaturase *FADS3*, the function of which is still not well understood but is known to be regulated by the presence of LCFA [52].

Other genes were annotated as having ‘*mitochondrial*’ functions including carnitine palmitoyl transferase (*CPT1B*), cytochrome oxidase *COX7C* and the calcium-binding mitochondrial solute carrier *SLC25A25*, all of which were down-regulated, whereas two orthologs of *COX7B* were both up-regulated. The suppressor of cytokine signalling *SOCS2* and insulin-like growth factor binding protein *IGFBP1*, both down-regulated, attenuate growth hormone (*GH*) signalling under fasting conditions [53]. Although LP is not a period of fasting, voluntary feed intake has been shown to decline in dairy cattle as parturition approaches, especially during the final week prepartum [8]. A shift towards earlier usage of labile body reserves in Fert- animals during LP was supported by our results, highlighting an aberration in energy metabolism that warrants further study. Further support for this was found with lipin 1 (*LPIN1*), down-regulated, which is involved in regulation of lipid metabolism by formation of a transcriptional regulation complex with peroxisome proliferator-activated receptor (PPAR) pathway genes *PPARA* and *PGC-1A* [54]. Circulating NEFA and glucose concentrations were not different at this time-point, however (see Fig. 1 and [17]).

### Early lactation

#### ‘Immune and inflammatory’ related

Alpha-1-acid glycoprotein 1 (*AGP1*; also called orosomucoid-1 (*ORM1*)) is a key gene involved in immune and inflammation regulation and response. AGP1 was down-regulated in liver at EL. This gene was the third most abundant transcript at EL using mean FPKM values. Functionally, *AGP* is an acute-phase plasma protein. In dairy cows, concentrations in blood increase with the severity of uterine bacterial infection and decline during uterine involution [55]. This is consistent with previous observations of the more favourable early postpartum uterine health status in Fert + cows compared with Fert- cows [19]. Additional genes involved in the acute phase response that were down-regulated included serum amyloid A3 (*SAA3*) and mammary serum amyloid A3.2 (*MSAA3.2*). *SAA3* expression is induced in endometrial epithelial cells in response to challenge with *Escherichia coli* [56]. Chemokines that were down-regulated include *CCL3, CCL4*, *CCL21* and *CXCL10*. These chemokines are known to play a functional role in the inflammatory response [57, 58]. Mannose-binding lectin (*MBL1*), also down-regulated, is important in bacterial recognition and innate immunity in cattle. A study using quantitative PCR assays reported elevated hepatic *MBL1* mRNA levels in cows with clinical mastitis compared with healthy cows, leading to the conclusion that *MBL1* may contribute to resistance to bacterial infection [59].

Activins, inhibins and follistatins are classically known for their role in regulating the synthesis, release and bioactivity of follicle stimulating hormone. These proteins are also involved in inflammation and immune response in many tissues. Inhibin E (*INHBE*), which is the precursor of activin bE, was found to be up-regulated at EL in liver. Activin bE has a high level of similarity to activin A, and can also bind its targets [60]. Activin A is well established as a pro-inflammatory mediator at lower levels of expression, but once inflammation is established and activin A levels rise, an inhibitory effect is seen [61]. Activins are bound by the binding proteins alpha-2-macroglobulin and follistatins. We found the follistatins *FST* and *FSTL1* up-regulated at EL. These genes are implicated in inhibition of activins, and therefore might allow inflammation to proceed [61]. There may be an interesting feedback system operating given that we have found both the potentially anti-inflammatory agent *INHBE* and its follistatin regulators up-regulated at EL.

Three genes annotated as tumour necrosis factor receptors were up-regulated (*TNFSR6D*, *TNFSR10B*, *TNFSR12A*). These genes are involved in regulating inflammation, in particular during the induction of apoptosis [62]. Multiple S100A gene family members known to play multiple roles in inflammatory processes were down-regulated in Fert + animals (*S100A4*, *S100A8*, *S100A9*, *S100A12 and S100A13*). In particular, the calgranulins *(S100A8, S100A9*, and *S100A12)* combine to form calprotectin, which is anti-inflammatory and reduces oxidative damage by reactive oxygen species (ROS) in hepatocytes [63]. Also of note, the superoxide dismutase *SOD2* was up-regulated. SOD2 is a mitochondrial anti-oxidant and is a nucleoid constituent with mitochondrial DNA (mtDNA) to protect against damage by ROS that might otherwise cause mitochondrial dysfunction [64]. Collectively, these findings suggest that the Fert + cows had greater antioxidant capacity during early lactation, which is particularly important at this time to combat increased metabolism-related production of ROS (e.g. oxidation of fatty acids).

#### ‘Metabolism, lipid and carbohydrate’ related

We found genes that suggested increased potential for gluconeogenesis at EL in liver. All of the genes discussed herein were up-regulated unless otherwise specified. Phosphoglycomutase (*PGM3*) converts glucose-1-phosphate to glucose-6-phosphate in glycogenolysis, facilitating conversion of stored glycogen to glucose if required [65]. Asparagine synthase (*ASNS*) converts aspartate to asparagine, which can then be converted to oxoaloacetate (the rate limiting compound in gluconeogenesis) for use in the citric acid cycle [66]. Further on in that cycle, succinyl-CoA is converted to succinate by succinyl-CoA synthetase (*SUCLA2*). Serine is a glucogenic amino-acid [67]; the final step of serine production from phosphoserine is controlled by the enzyme phosphoserine phosphatase (*PSPH*).

In muscle, annotation terms *‘lipid lipoprotein*’, ‘*metabolic process*’ and ‘*amine process*’ all had multiple up-regulated DE genes involved in glucose and fatty acid metabolism. These genes included: serine dehydratase (*SDS*), which is involved in serine metabolism; phenylethanolamine N-methyltransferase (*PNMT*), which is involved in norepinephrine conversion to epinephrine; low-density lipoprotein receptor (*LDLR*); protein kinase gamma *PRKAG3*; pyruvate kinase *PKM2*; phosphoglycerate mutase *PGMA2*; pyruvate dehydrogenase *PDP2*; the peroxisome proliferator-activated receptor co-activator *PPARGC1A*; *ECHDC2*; enolase *ENO3*; glycerol-3-phosphate dehydrogenase 1 (*GDP1*) and lactate dehydrogenase (*LDHA*). A recent review highlighted the action of *PPARGC1A* in inducing a variety of genes under fasting conditions including estrogen related receptor gamma *ESRRG*, which we found up-regulated [53]. We also found glucose transporters *GLUT1* and *GLUT4* (or *SLC2A1* and *SLC2A4*) up-regulated. Related genes found down-regulated include acyl-CoA synthase *ACSL4*, apolipoproteins *APOA2* and *APOD* and monoamine oxidase MAOA.

### Mid-Lactation

#### ‘Immune and inflammatory’ related

Multiple acute phase protein genes such as *SAA1*, *SAA2* and *SAA3*, haptoglobin (*HP*) and glutathione peroxidises *GPX2* and *GPX3* were all down-regulated in liver. Similarly in muscle, acute phase proteins *HP*, *SAA1*, *AGP* and fibrinogens *FGA*, *FGB* and *FGG* were all down-regulated. These genes have been shown to have greater serum protein expression in cows during disease states [68]. These results once more indicate an inherently different ability to regulate inflammation between cows with divergent genetic merit for fertility.

#### ‘Metabolism, lipid and carbohydrate’ related

Genes with annotated functions related to ‘*lipids*’ confirmed the quantitative real-time PCR work of Cummins and colleagues [18] undertaken on some of the same animals used in this study. In particular, insulin-like growth factor 1 (*IGF-1*) was up-regulated in liver. Low circulating insulin during early lactation causes uncoupling of the GH-IGF axis. This results in partitioning of glucose away from insulin-responsive muscle and adipose tissue towards the mammary gland, which is not insulin-responsive [69]. As previously outlined [17, 18], greater hepatic *IGF-1* expression during ML is likely a key mechanism responsible for body reserve repletion in Fert + cows.

Other related genes, all of which were down-regulated in liver, included apolipoproteins *APOA1* and *APOA4*, the cytochrome p450s *CYP11A1* and *CYP1B1*, previously reported fatty acid binding proteins *FABP3* and *FABP4*, *PSPH*, prostaglandin synthases *AKR1C1*, *PTGF2SL* and *PTGDS* and sulfotransferase *SULT1E1.* Apolipoproteins are constituents of plasma lipoproteins, and fatty-acid binding proteins transport lipids for beta-oxidation in mitochondria, especially LCFA (24) [51]. Up-regulated genes in this group included acetyl-CoA carboxylase *ACACA*, acyl-CoA synthase *ACSS2*, ceramide synthase *CERS6*, fatty acid synthase *FASN*, synthase and reductase of 3-hydroxy-3-methylglutaryl-CoA *HMGCS1* and *HMGCSR*, and squalene epoxidase *SQLE*.

Finally, in muscle we observed multiple DE genes with annotation terms including ‘*lipid synthesis*’ and ‘*glucose metabolism*’. Genes of interest that were down-regulated include apolipoproteins *APOA5* and *APOH*, *FABP4*, glutamate ammonia ligase (*GLUL*), and diacylglycerol O-acyltransferase 2 (*DGAT2*). All of these genes appear to be involved in facilitating greater BCS in Fert + cows, although the specific mechanisms have yet to be elucidated. Overall, the trend seems to indicate that genes involved in fatty acid synthesis pathways are up-regulated in liver tissue of Fert + cows at ML, and that genes involved in lipid synthesis in muscle are increased in Fert- animals at the final timepoint.

## Conclusion

The requirement for greater gluconeogenic capacity in early lactation is a well-studied phenomenon, as is the concomitant change in immune function and inflammation [15]. Here we demonstrate that animals genetically and phenotypically divergent for fertility traits exhibit transcriptomic differences in liver and muscle related to both of these processes at three important time-points during the gestation-lactation cycle. These signals are found across every time-point and in both tissues, indicating that this may be a systemic response in these cows. The findings reported here, and the studies of others [8, 15, 49, 70], indicate that immune regulation and inflammatory processes are functional at many stages of the gestation-lactation cycle, and are important for dairy cow fertility and reproductive efficiency. In particular, our results indicate that Fert- cows are less capable of regaining control of inflammation following the onset of lactation. Evidence at the mid-lactation time-point where key metabolism and fertility-related genes such as *IGF-1* were differentially regulated supports the idea that both immune/inflammatory and metabolic processes are fundamental to the phenotypic divergence in our model. It is plausible that these processes are linked. It is also plausible that an increased burden of ROS coupled with a chronic inflammatory state (therefore potentially explaining the continuous expression of BOLA genes into ML) results in Fert- cows expending greater resources trying to effectively maintain/regain body condition compared with Fert + cows.

## Abbreviations

BAM, binary alignment/map; BCS, body condition score; DE, differentially expressed; EBV, estimated breeding value; EL, early lactation; LP, late pregnancy; ML, mid-lactation; NEB, negative energy balance; NEFA, non-esterified fatty acids; ROS, reactive oxidation species; SAM, sequence alignment/map

## Additional files

Additional file 1: Table S1. Liver Differentially Expressed Gene Data; XLSX workbooks with 6 sheets: (i) “1N_vs_1P.sig”, (ii) “2N_vs_2P.sig”, (iii) “3N_vs_3P.sig” contain differentially expressed genes between FertN and FertP cows at time-points 1,2,3 respectively, (iv) “in_all_TP” are genes found DE in all time-points, (v) fpkms_X are the full FPKMs for each gene in the tissue, (vi) “DAVID_annotation_LP_EL_ML_X” are annotations from DAVID per time-point, specifying each term found significantly enriched for genes, and the DE genes with an arrow to indicate direction of dysregulation, down being lower expression in FertP. (XLSX 15 mb) Additional file 2: Table S2. Muscle Differentially Expressed Gene Data respectively; XLSX workbooks with 6 sheets: (i) “1N_vs_1P.sig”, (ii) “2N_vs_2P.sig”, (iii) “3N_vs_3P.sig” contain differentially expressed genes between FertN and FertP cows at time-points 1,2,3 respectively, (iv) “in_all_TP” are genes found DE in all time-points, (v) fpkms_X are the full FPKMs for each gene in the tissue, (vi) “DAVID_annotation_LP_EL_ML_X” are annotations from DAVID per time-point, specifying each term found significantly enriched for genes, and the DE genes with an arrow to indicate direction of dysregulation, down being lower expression in FertP. (XLSX 15 mb) Additional file 3: Table S3. Sequencing Metrics; total reads, aligned reads and uniquely mapped reads per sample in each tissue with mean and standard deviation for each category. (XLSX 11 kb) Additional file 4: Figure 1(a-f). Correlation plots of gene expression values in FPKM for each time-point versus all others per tissue. (ZIP 324 kb)
