# Supplementary figures and images for: Transcriptomics of liver and muscle in Holstein cows genetically divergent for fertility highlight differences in nutrient partitioning and inflammation processes
Source: BMC Genomics. 2016 Aug 11;17:603. doi: 10.1186/s12864-016-2938-1 (PMC4982134; doi:10.1186/s12864-016-2938-1)

# Gene Expression Correlation of TP1 vs TP2 in Liver

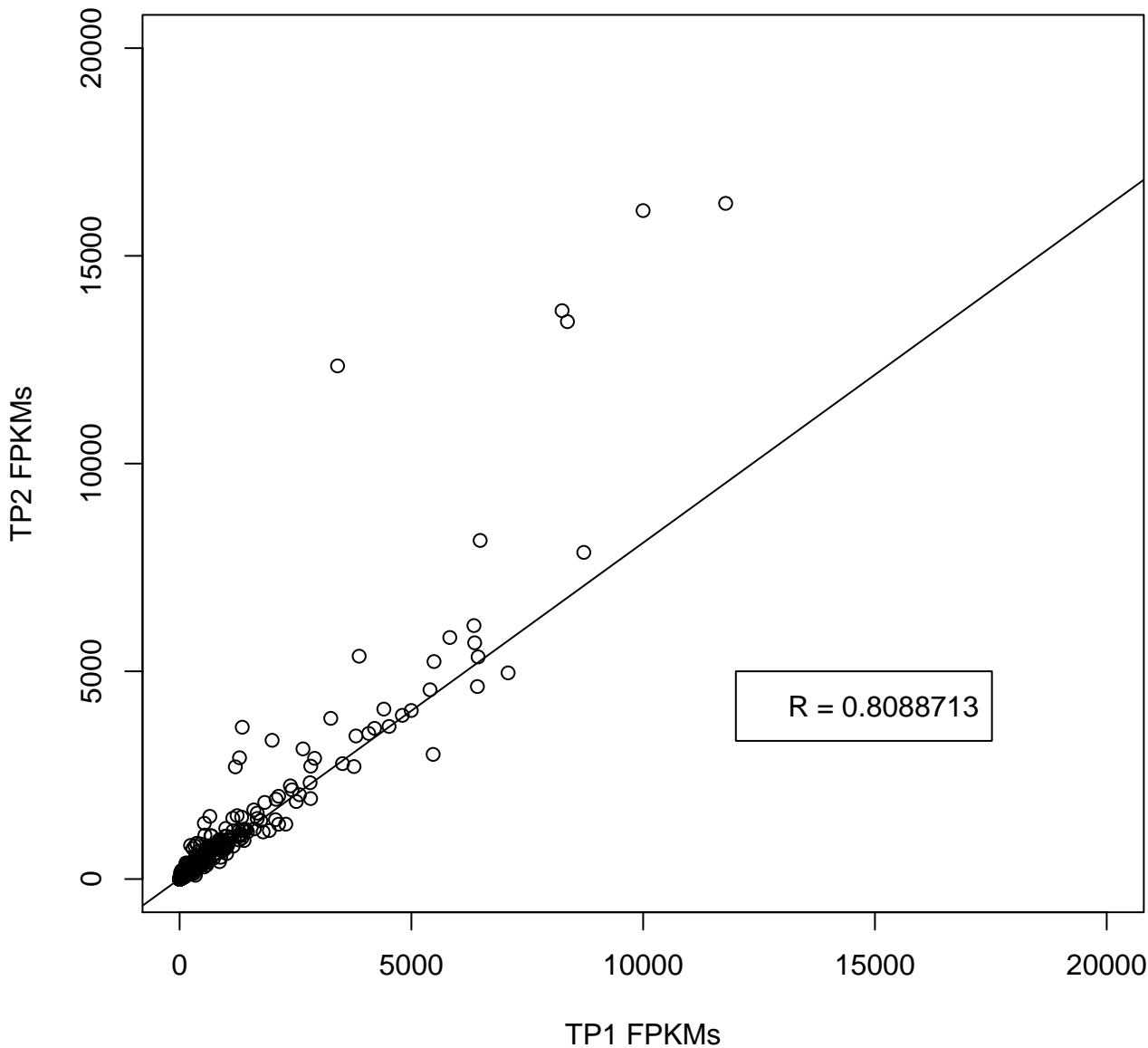

Supplement: Additional file 4: Figure 1(a-f). — Correlation plots of gene expression values in FPKM for each time-point versus all others per tissue. (ZIP 324 kb) [file 12864_2016_2938_MOESM4_ESM.zip › Liver_TP-1_vs_2_corrplot.pdf]

# Gene Expression Correlation of TP1 vs TP3 in Liver

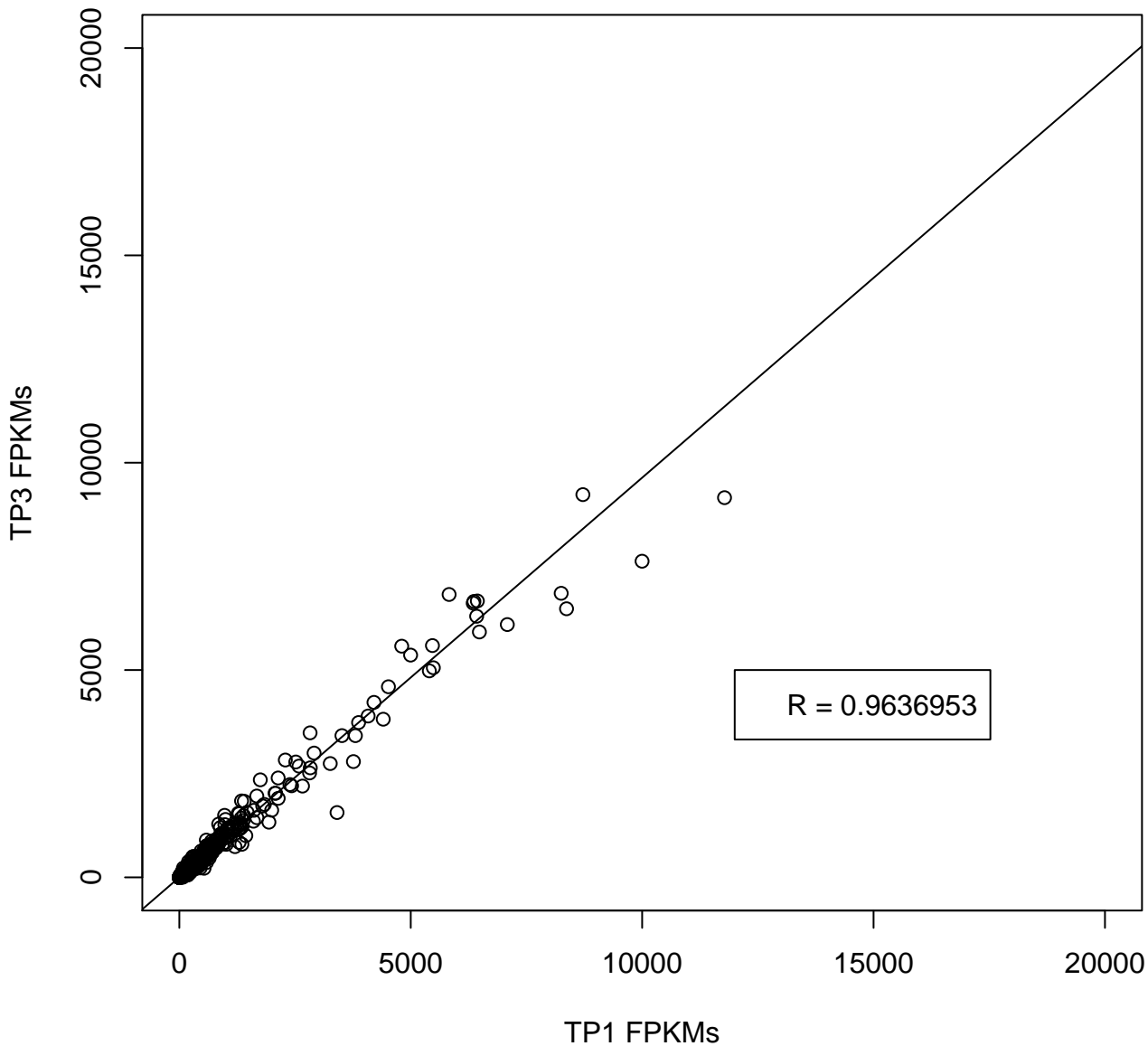

Supplement: Additional file 4: Figure 1(a-f). — Correlation plots of gene expression values in FPKM for each time-point versus all others per tissue. (ZIP 324 kb) [file 12864_2016_2938_MOESM4_ESM.zip › Liver_TP-1_vs_3_corrplot.pdf]

# Gene Expression Correlation of TP2 vs TP3 in Liver

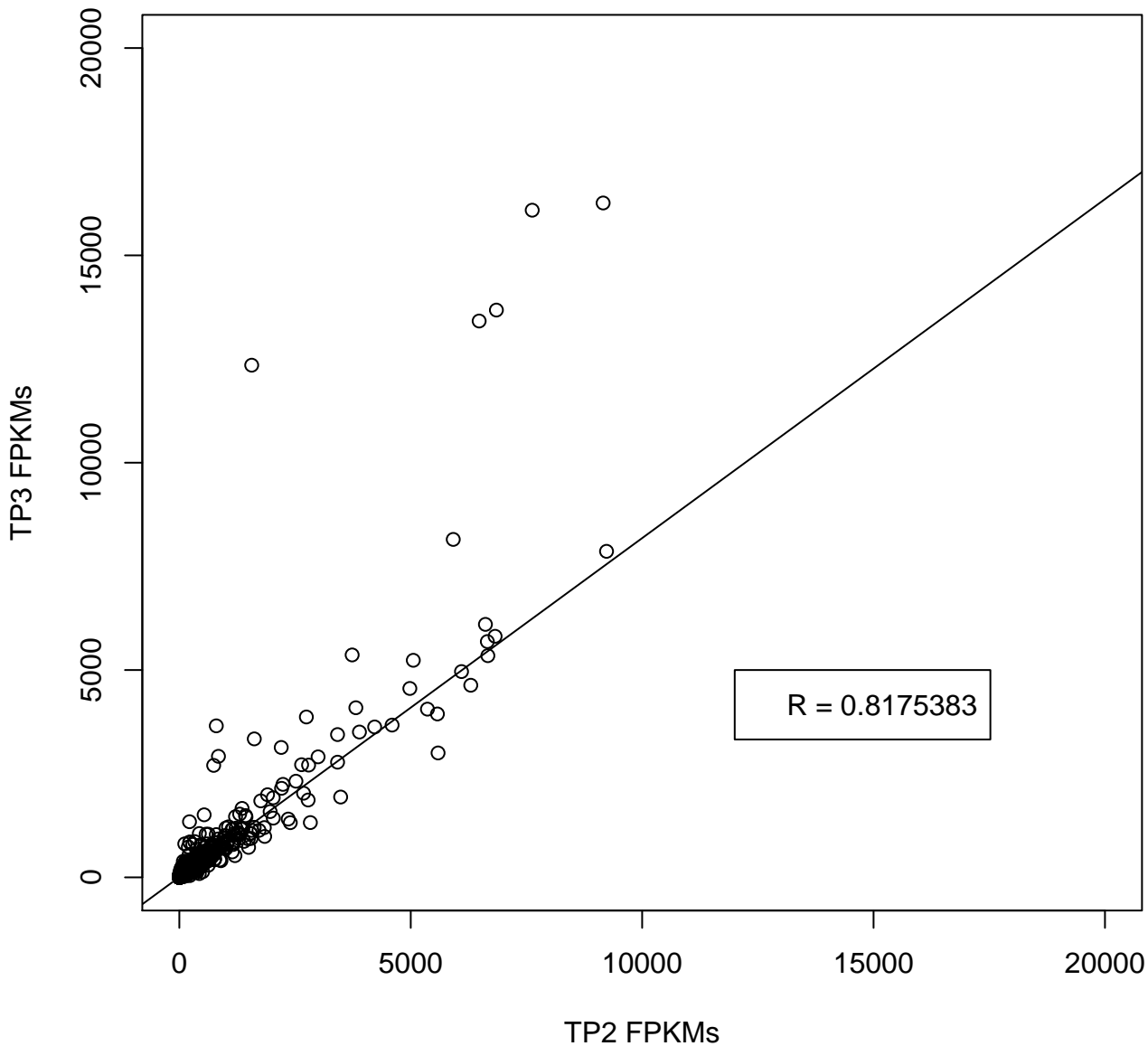

Supplement: Additional file 4: Figure 1(a-f). — Correlation plots of gene expression values in FPKM for each time-point versus all others per tissue. (ZIP 324 kb) [file 12864_2016_2938_MOESM4_ESM.zip › Liver_TP-2_vs_3_corrplot.pdf]

# Gene Expression Correlation of TP1 vs TP2 in Muscle

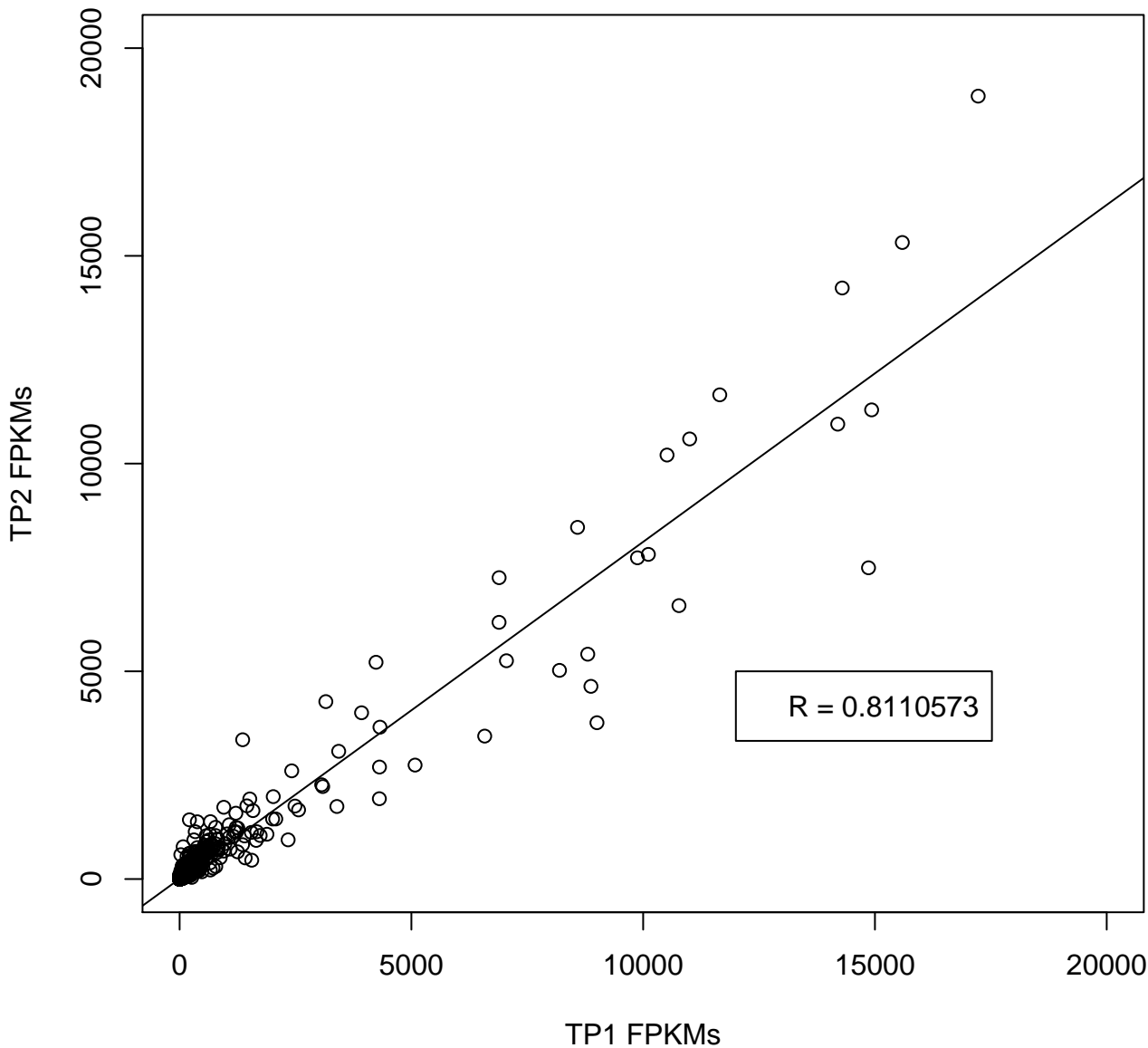

Supplement: Additional file 4: Figure 1(a-f). — Correlation plots of gene expression values in FPKM for each time-point versus all others per tissue. (ZIP 324 kb) [file 12864_2016_2938_MOESM4_ESM.zip › Muscle_TP-1_vs_2_corrplot.pdf]

# Gene Expression Correlation of TP1 vs TP3 FPKMs in Muscle

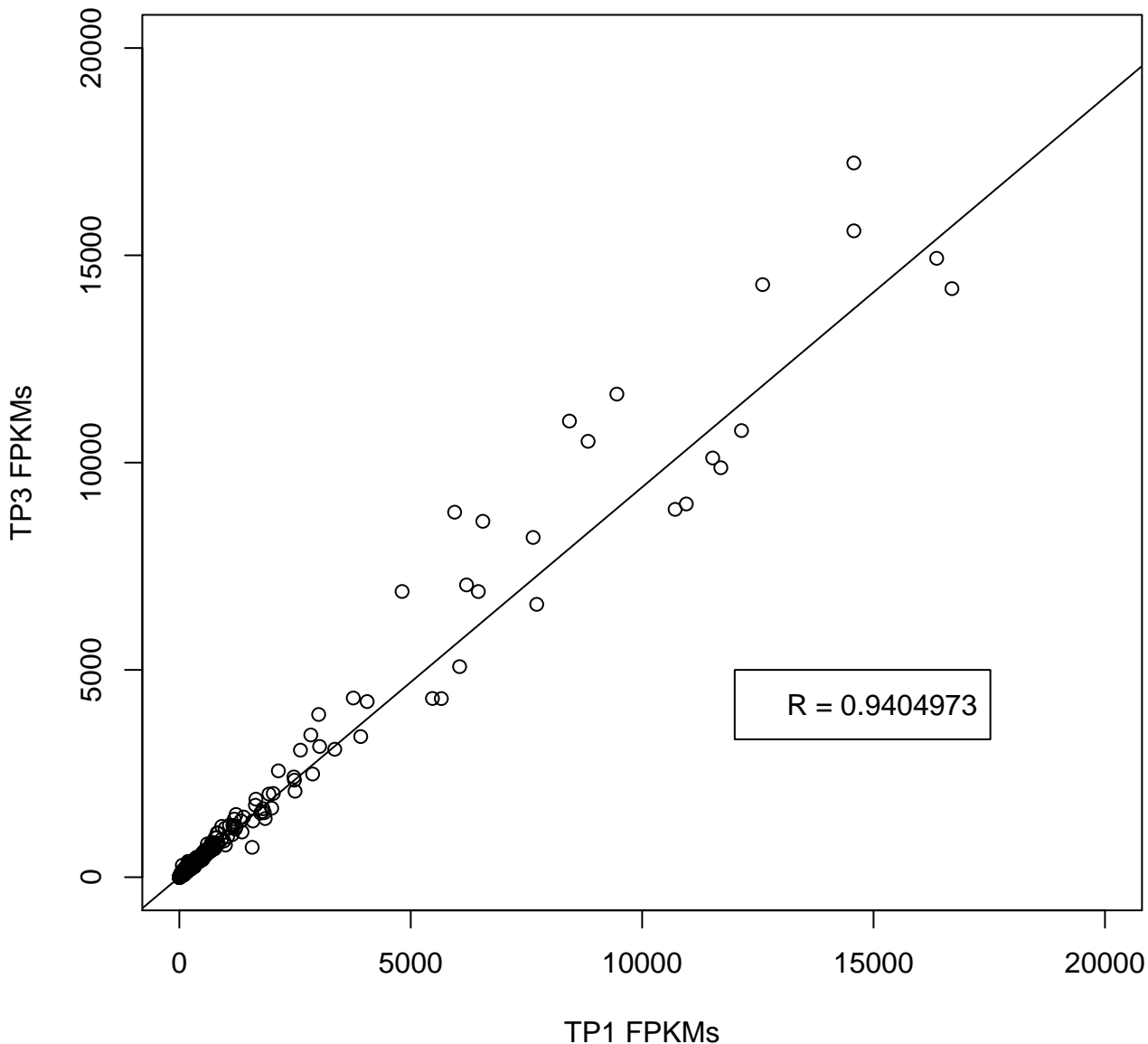

Supplement: Additional file 4: Figure 1(a-f). — Correlation plots of gene expression values in FPKM for each time-point versus all others per tissue. (ZIP 324 kb) [file 12864_2016_2938_MOESM4_ESM.zip › Muscle_TP-1_vs_3_corrplot.pdf]

# Gene Expression Correlation of TP2 vs TP3 in Muscle

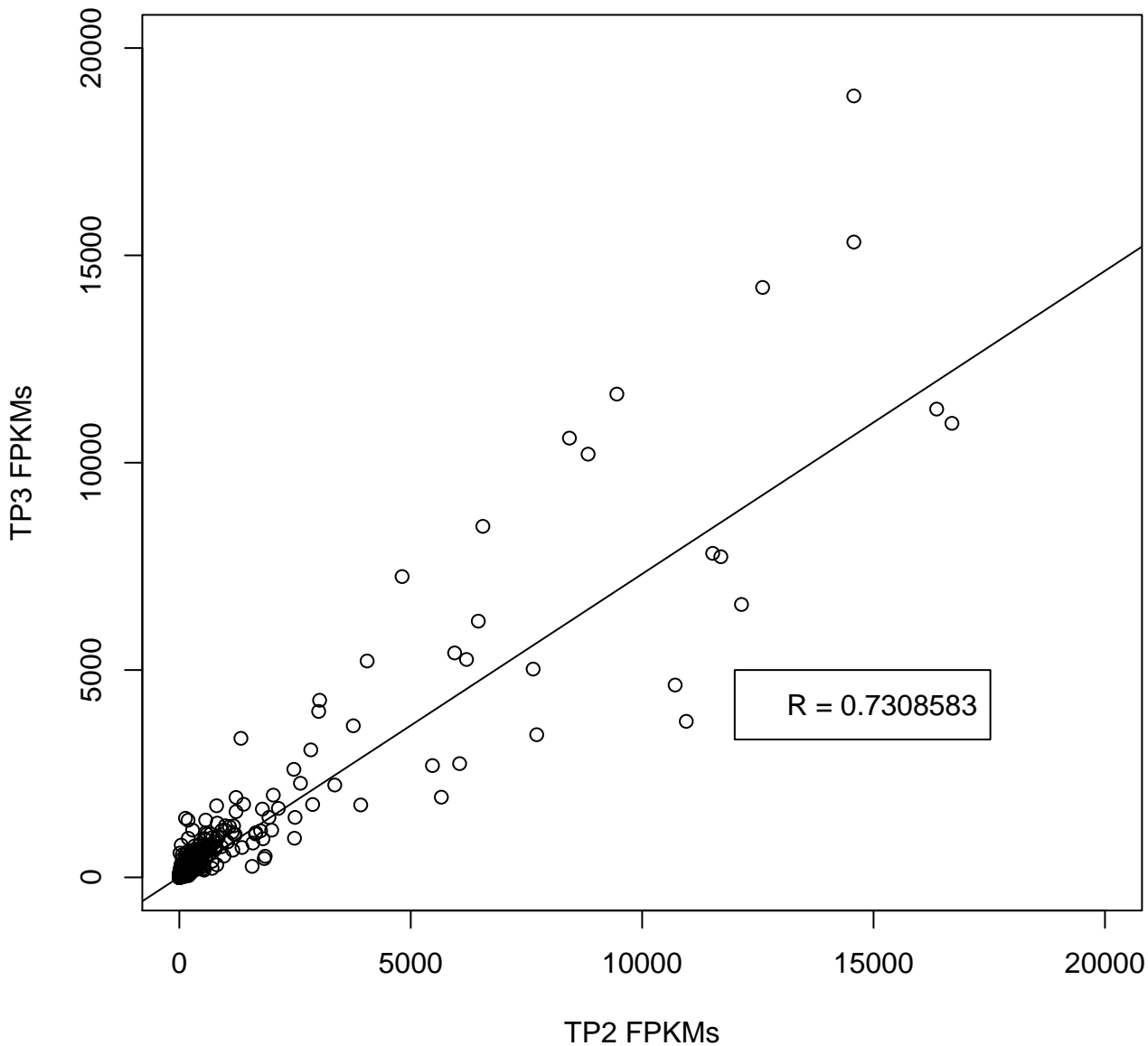

Supplement: Additional file 4: Figure 1(a-f). — Correlation plots of gene expression values in FPKM for each time-point versus all others per tissue. (ZIP 324 kb) [file 12864_2016_2938_MOESM4_ESM.zip › Muscle_TP-2_vs_3_corrplot.pdf]
